# Molecular Landscape and Therapeutic Strategies against Colorectal Cancer

**DOI:** 10.3390/cancers16081551

**Published:** 2024-04-18

**Authors:** Aakash Patel, Pat Gulhati

**Affiliations:** 1 Division of Medical Oncology, Department of Medicine, Robert Wood Johnson Medical School, Rutgers University, New Brunswick, NJ 08901, USA; 2 Rutgers Cancer Institute of New Jersey, Rutgers University, New Brunswick, NJ 08901, USA

**Keywords:** colorectal cancer, targeted therapy, immunotherapy, next generation sequencing, young-onset colorectal cancer

## Abstract

**Simple Summary:**

Colorectal cancer (CRC) is the second leading cause of cancer deaths worldwide. Genetic alterations promote cancer development and its spread to distant sites. Medications that counteract the effects of these alterations/mutations, called targeted therapies, are used to treat CRC. Immunotherapy, another class of medications that promotes immune system mediated recognition and destruction of cancer cells, is used for treatment of a small subset of CRC patients. Here we review the current state of knowledge and ongoing research into biomarkers, targeted therapy, and immunotherapy for CRC treatment.

**Abstract:**

Colorectal cancer (CRC) is the second leading cause of cancer deaths worldwide. Although the overall incidence of CRC is decreasing, the incidence of young-onset CRC, characterized by a diagnosis of CRC before age 50, is increasing. Outcomes for CRC patients are improving, partly due to comprehensive molecular characterization of tumors and novel therapeutic strategies. Advances in genomic and transcriptomic analyses using blood- and tumor-tissue-based sequencing have facilitated identification of distinct tumor subtypes harboring unique biological characteristics and therapeutic vulnerabilities. These insights have led to the development and incorporation of targeted therapies and immunotherapy in CRC treatment. In this review, we discuss the molecular landscape and key oncogenes/tumor suppressors contributing to CRC tumorigenesis, metastasis, and therapeutic resistance. We also discuss personalized therapeutic strategies for subsets of CRC patients and provide an overview of evolving novel treatments being evaluated in clinical trials.

## 1. Introduction

Colorectal cancer (CRC) is the third most common cancer in the US and the second most common cause of cancer specific mortality worldwide [1,2]. Risk factors for CRC include a variety of modifiable factors such as diet, alcohol, and tobacco use, along with non-modifiable risk factors such as sporadic or inherited genetic alterations [3]. While the overall incidence and mortality of CRC has been decreasing for decades, there has been an increase in CRC incidence in adults under age 50 (young-onset CRC) and also a slight increase in mortality in this subgroup since the early 2000s [1,4]. In addition, researchers have identified differences in CRC outcomes based on tumor biology, such as primary colonic tumor sidedness and colonic versus rectal cancers [5,6]. These initial insights provided a foundation for researchers to further investigate the molecular landscape of CRC. In this review, we discuss genomic and transcriptomic analysis techniques that facilitated identification of driver molecular alterations and biomarkers in CRC. We also discuss the implementation of targeted therapies and immunotherapies in the personalized treatment of CRC patients.

## 2. Molecular Diagnostic Techniques

### 2.1. Genomic Sequencing

There are a multitude of molecular testing methods available to detect molecular alterations and determine targeted therapy and immunotherapy candidacy for CRC patients. Clinical guidelines, such as those provided by the American Society of Clinical Oncology and the National Comprehensive Cancer Network, recommend genomic sequencing in patients with metastatic or advanced CRC, using next generation sequencing (NGS) [7,8]. Tumor tissue NGS is performed directly on biopsy samples or surgical specimens, while blood based NGS testing assesses circulating tumor DNA (ctDNA). Tumor tissue NGS is preferred when feasible compared to ctDNA analysis since ctDNA may not be detectable in up to 15% of patients due to factors such as tumor burden and location, among others [9]. For example, an analysis of plasma ctDNA NGS relative to tissue NGS of peritoneal metastases in patients with gastrointestinal malignancies found a concordance of only 18% [10]. Nonetheless, ctDNA results are available sooner than tumor tissue NGS, and can expedite treatment decisions and clinical trial enrollment. In a study comparing gastrointestinal cancer trial enrollment in two large studies in Japan, median time to obtain tissue NGS results after sample collection was 19 days relative to 7 days for blood ctDNA NGS. In addition, there was also a delay in obtaining tissue biopsies relative to blood draws. Overall, these delays resulted in a total median time for trial enrollment with tissue NGS of 33 days relative to 11 days for blood NGS [11]. A unique benefit of ctDNA analysis is the potential role for minimal residual disease (MRD) detection following curative intent treatments. In CRC patients who underwent curative intent surgery without signs of macroscopic disease, ctDNA-based MRD analysis that included serial surveillance analysis demonstrated a specificity of 100% and a sensitivity of 91% for disease recurrence, relative to a specificity of 80.7% and sensitivity of 35% with CEA analysis [12].

### 2.2. Transcriptomics

The addition of transcriptomic data to genomic analysis has allowed researchers to identify distinct CRC molecular subtypes known as consensus molecular subtypes (CMS) [13]. CMS1 is characterized by immune activation and microsatellite instability-high (MSI-H), suggesting susceptibility to immunotherapy. The other characterized CMS subtypes provide a basis for the development of future targeted therapies and are distinguished as follows: CMS2 demonstrates wingless and Int-1 (WNT) and MYC activation, CMS3 demonstrates metabolic dysregulation, and CMS4 is notable for transforming growth factor beta (TGFß) activation with angiogenesis and stromal invasion [14].

### 2.3. Proteomics

Proteomic data has been used to refine existing genomic profiles of CRC patients. Combined proteogenomic analysis of CRC tumors by The Cancer Genome Atlas (TCGA) has shown that mRNA transcript levels do not correlate with protein levels, and chromosome 20q amplification results in the greatest change in both mRNA and protein abundance [15]. Five distinct proteomic subtypes of CRC have been identified, labeled as proteomic subtypes A through E. Proteomic subtypes B and C overlap with the TCGA MSI subtype, while the other three subtypes are associated with chromosomal instability [15]. Further, phosphoproteomic analysis in prospectively collected paired tumor and normal adjacent tissues has identified Rb phosphorylation as an oncogenic driver and potential therapeutic target [16]. In this study, multi-omics analysis incorporating multiple existing classification systems such as the aforementioned CMS and proteomic subtypes led to the identification of three unified multi-omics subtypes (UMS) labeled as MSI, CIN, and mesenchymal [16]. In the UMS MSI subtype, an increase in glycolysis enzymes was associated with decreased CD8^+^ T cell tumor infiltration, suggesting potential for therapeutic inhibition of glycolytic enzymes in MSI tumors that are resistant to immunotherapy [16]. Additional proteomic studies have demonstrated that the facilitates chromatin transcription (FACT) complex promotes 5-Fluorouracil (5-FU) resistance in dMMR CRC tumors by recruiting DNA damage repair proteins, including APE1, in the setting of 5-FU-induced DNA damage [17]. FACT is elevated in multiple tumor types and is associated with poor survival [18,19]. Curaxins are small molecules that trap the FACT complex within chromatin, leading to p53 activation and NF-κB inhibition, resulting in anti-tumor activity [20].

### 2.4. Multi-Omics

Multi-omics analysis of CRC tumors has facilitated novel immunologic and microbiologic insights in CRC. An immune signature panel of 20 genes encompassing type 1 T helper cell and cytotoxic immune activation, termed the Immunologic Constant of Rejection (ICR), was found to be associated with overall survival (OS) independent of MSI and CMS [21]. Through deep T cell receptor sequencing, the ICR is hypothesized to derive prognostic relevance by capturing the presence of tumor-enriched clonal T cell populations [21]. 16S bacterial rRNA gene sequencing has identified that relative abundance of specific microbes can impact prognosis in CRC. For instance, Ruminococcus bromii has been associated with favorable outcomes, while Fusobacterium nucleatum is associated with worse outcomes [21]. Combining the ICR and microbiome signature into a composite score, mICRoScore, can identify patients with improved survival in CRC [21]. Single-cell RNA sequencing has identified that while both deficient mismatch repair (dMMR) and MMR proficient tumors have myeloid inflammatory foci, only dMMR tumors have multicellular foci of CXCR3 chemokine positive cells [22]. Insights such as these can facilitate advances in prognostication and therapeutic development in CRC.

## 3. Potentially Targetable Genomic Alterations

### 3.1. VEGF

**Figure 1:**
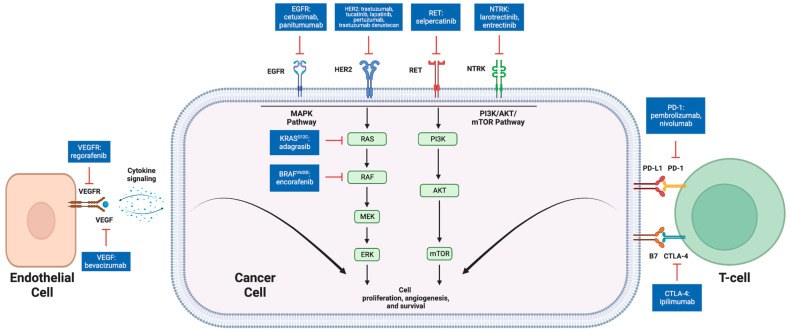
Select targeted therapy and immunotherapy agents used in CRC treatment. Created with BioRender.com.

**Figure 2:**
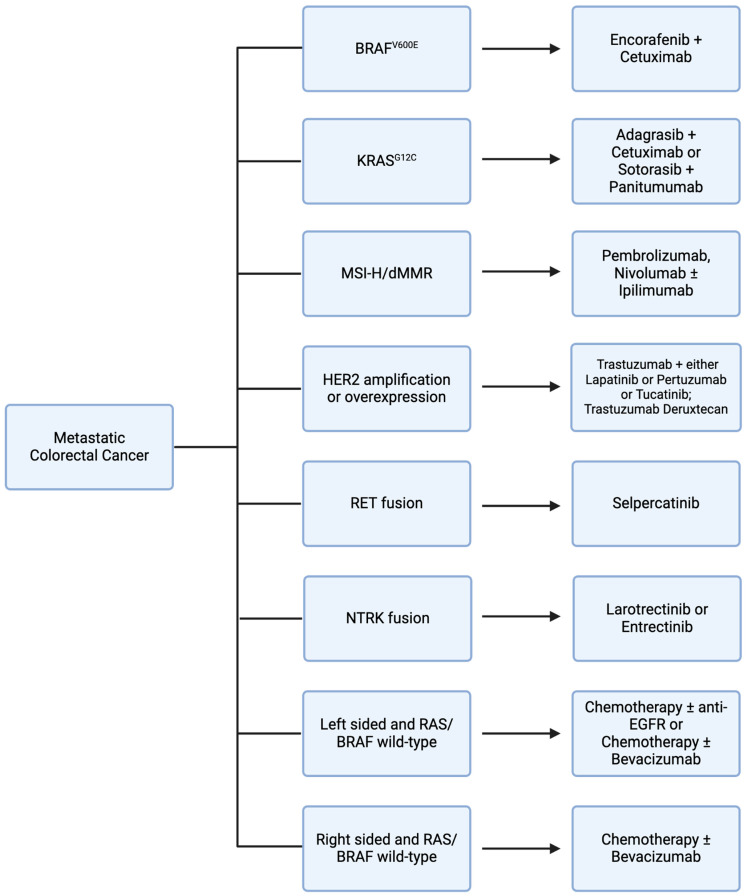
Flowchart depicting systemic therapy options in metastatic colorectal cancer based on tumor mutational status/sidedness. Created with BioRender.com.

Vascular endothelial growth factor (VEGF) is a signaling protein that promotes angiogenesis and cellular proliferation when bound to VEGF receptors on endothelial cells [23] (Figure 1). Bevacizumab is an anti-VEGF humanized monoclonal antibody used in first-line metastatic CRC (mCRC) treatment in conjunction with fluorouracil-based chemotherapy [24,25] (Table 1) (Figure 2). The role of maintenance bevacizumab in mCRC was investigated in CAIRO3, a study of capecitabine plus bevacizumab maintenance versus observation in patients with disease stabilization/response to first-line capecitabine, oxaliplatin, and bevacizumab. In both groups at progression, patients again received capecitabine, oxaliplatin, and bevacizumab until second progression. The primary endpoint, median time to second progression, was 8.6 months (95% CI 7.0–10.1 months) in the observation group compared to 11.6 months (95% CI 10.0–13.3 months) in the capecitabine plus bevacizumab maintenance group with a hazard ratio of 0.64 (95% CI 0.53–0.77) [26]. Therapeutic resistance to VEGF inhibition is thought to be mediated by tumor microenvironment changes and alternative angiogenic pathways, such as HGF/Met and SDF-1/CXCR-4, among others; however, studies demonstrate that there is efficacy in the continuation of bevacizumab in subsequent line treatment [27]. The ML18147 trial supports continuation of bevacizumab in second-line treatment for mCRC refractory to first-line chemotherapy with bevacizumab. In this phase III trial of 820 mCRC patients with progression on first-line bevacizumab plus chemotherapy, patients were treated with second-line chemotherapy with or without bevacizumab. Median OS was 11.2 months (95% CI 10.4–12.2 months) for the chemotherapy plus bevacizumab group compared to 9.8 months (95% CI 8.9–10.7 months) for the chemotherapy alone group with a hazard ratio of 0.81 (95% CI 0.69–0.94). In the bevacizumab group relative to the chemotherapy alone group, several toxicities were noted, including bleeding, gastrointestinal perforation, and venous thromboembolism [28].

Regorafenib was the first multiple receptor tyrosine kinase inhibitor, which includes inhibition of the VEGF receptor, to demonstrate a survival benefit in mCRC that had progressed on standard-of-care therapy with fluoropyrimidines, oxaliplatin- and irinotecan-based chemotherapy, an anti-VEGF agent, and anti-EGFR therapy (if RAS/BRAF wild-type). In the phase III CORRECT trial, 760 mCRC patients with previous treatment/progression were randomized to regorafenib versus placebo. Median OS was 6.4 months in the regorafenib group relative to 5.0 months in the placebo group with a hazard ratio of 0.77 (95% CI 0.64–0.94). The most common adverse events included hand–foot cutaneous reactions, fatigue, diarrhea, and hypertension [29] (Table 1). These encouraging results led to FDA approval of regorafenib for refractory mCRC in September 2012 [30]. More recently, a narrower-spectrum VEGF-receptor-targeted therapy, fruquintinib, has been studied in refractory mCRC and obtained FDA approval in November 2023. FRESCO-2 was a global phase III study of 691 patients with previously treated mCRC randomly assigned in a 2:1 ratio to receive VEGF receptor inhibitor fruquintinib versus placebo, respectively. Median OS was 7.4 months (95% CI 6.7–8.2 months) in the fruquintinib group versus 4.8 months (95% CI 4.0–5.8 months) in the placebo group with a hazard ratio of 0.66 (95% CI 0.55–0.80). The most common grade 3 or greater adverse events in the fruquintinib group included hypertension, asthenia, and hand–foot syndrome [31] (Table 1).

### 3.2. EGFR

Epidermal growth factor receptor (EGFR) is a receptor tyrosine kinase involved in downstream activation of multiple cellular proliferation pathways, such as the MAPK and PI3K pathways, which leads to tumorigenesis [32,33]. Cetuximab and panitumumab are anti-EGFR monoclonal antibodies used in the treatment of mCRC (Figure 1). Notably, constitutively activating mutations in genes that encode downstream signaling proteins of the RAS mitogen activated protein kinase (MAPK) pathway, such as KRAS and BRAF, decrease sensitivity to EGFR inhibitors [34,35]. In the PRIME trial of fluorouracil, leucovorin, and oxaliplatin with or without panitumumab, among patients with wild type KRAS, combined chemotherapy and panitumumab demonstrated a progression-free survival (PFS) of 9.6 months relative to 8.0 months with chemotherapy alone with a hazard ratio of 0.80 (95% CI 0.66–0.97). In contrast, among patients with KRAS mutations, combined chemotherapy and panitumumab demonstrated a PFS of 7.3 months relative to 8.8 months with chemotherapy alone with a hazard ratio of 1.29 (95% CI 1.04–1.62) [36] (Table 1). In addition, right-sided colon tumors, which have increased prevalence of KRAS and BRAF mutations and are associated with worse prognosis compared to left-sided colon tumors, respond poorly to EGFR inhibition [37]. In retrospective analysis of the CRYSTAL trial evaluating fluorouracil, leucovorin, and irinotecan (FOLFIRI) with or without cetuximab and the FIRE-3 trial comparing FOLFIRI with either cetuximab or bevacizumab, there was minimal efficacy noted with cetuximab among patients with right-sided colon tumors [38]. Thus, while EGFR inhibition is a mainstay in mCRC treatment, consideration must be given to mutational profiles and primary tumor sidedness when evaluating suitability of treatment regimens (Figure 2).

EGFR-inhibitor-acquired resistance is associated with the development of RAS mutant clonal cancer cell populations that regress upon EGFR inhibitor cessation, providing rationale for potential benefit from EGFR inhibitor rechallenge [39]. Cetuximab rechallenge with irinotecan as third-line therapy was assessed in a phase II single-arm study of 28 patients with RAS/BRAF wild-type mCRC previously treated with first-line irinotecan- and cetuximab-containing regimens and second-line oxaliplatin- and bevacizumab-containing regimens. The response rate with cetuximab rechallenge plus irinotecan was 21% (95% CI 10–40%). RAS mutations were noted by ctDNA in 48% of patients prior to rechallenge, and among patients with partial response there were no RAS mutations. Additionally, those with RAS wild type ctDNA had a median PFS of 4.0 months relative to 1.9 months for those with RAS mutant ctDNA with a hazard ratio of 0.44 (95% CI 0.18–0.98) [40]. In the phase II single-arm CHRONOS trial, patients with RAS wild-type mCRC previously treated with anti-EGFR regimens underwent ctDNA screening to guide EGFR rechallenge with panitumumab in the third-line setting. Of 52 screened patients, 31% carried a ctDNA RAS, RAF, or EGFR mutation and were excluded. Among the 27 patients that were treated with panitumumab, 30% achieved partial response and 63% achieved disease control [41]. Recently, researchers analyzed mutational signatures of RAS/BRAF/EGFR wild-type mCRC patients pooled from three large, randomized trials who were treated with EGFR inhibition and chemotherapy in the first-line setting and EGFR inhibition alone in the third-line setting, and also analyzed transcriptional changes in a CRC cell line resistant to chemotherapy plus cetuximab. Findings from this study suggest that transcriptional changes account for resistance to combination chemotherapy plus EGFR inhibition, whereas acquired MAPK mutations account for resistance to EGFR inhibition alone [42].

### 3.3. BRAF

BRAF is a gene encoding a protein kinase involved in MAPK signaling, which is involved in cellular proliferation and survival [43] (Figure 1). Mutations in BRAF are seen in up to 10% of CRC patients, 95% of which are BRAF V600E mutations [44]. BRAF V600E is a constitutively activating mutation, promoting uncontrolled cellular replication and carcinogenesis [44]. BRAF V600E mutant CRC is associated with right-sided primary tumors and worse prognosis compared to BRAF wild type CRC [45]. Among BRAF V600E mutant CRC patients, those additionally harboring dMMR have improved outcomes relative to patients with intact MMR [46].

Currently, RAF inhibitor encorafenib plus cetuximab is approved for previously treated BRAF V600E mutant mCRC based on the phase III BEACON trial [47] (Table 1). In this study, 665 patients with previously treated BRAFV600E mutant mCRC were treated with either a triplet (encorafenib, binimetinib, and cetuximab) or doublet (encorafenib and cetuximab) or control (cetuximab plus chemotherapy). Median OS was 9.3 months with both triplet and doublet therapy, compared with 5.9 months for the control group. In addition to a similar median OS, doublet therapy demonstrated fewer adverse events, with 65.8% grade 3 or greater adverse events with triplet therapy compared to 57.4% for doublet therapy [48,49]. With comparable efficacy to triplet therapy and a better toxicity profile, doublet (encorafenib plus cetuximab) has become the standard of care for previously treated BRAF V600E mutant mCRC (Figure 2).

The BREAKWATER trial is a phase III study with a planned enrollment of 870 treatment-naive patients with BRAF V600E mutant mCRC investigating the efficacy of BRAF inhibition in the first-line setting. Treatment arms include encorafenib plus cetuximab versus encorafenib plus cetuximab plus chemotherapy versus control chemotherapy with or without cetuximab [50]. BREAKWATER safety lead-in and preliminary efficacy data demonstrate acceptable safety profiles with encorafenib plus cetuximab plus chemotherapy and favorable overall response rates relative to historical outcomes with current first-line standard-of-care regimens [51].

Combinations of immunotherapy and BRAF inhibitor are being investigated in BRAF V600E mutant CRC in patients with and without dMMR/MSI-H [52,53,54,55]. Downstream to BRAF in the MAPK pathway are extracellular signal-regulated kinases 1/2 (ERK1/2), thought to be involved in the acquired resistance to BRAF inhibitor therapy [56]. Multiple phase I and phase II trials are evaluating treatment with ERK inhibitors alone or in combination with BRAF inhibitors [57]. One ongoing trial, NeoBRAF, is investigating BRAF inhibitors in the neoadjuvant and adjuvant setting, which supports ongoing attempts to apply targeted therapies in earlier stages of disease [58] (Table 2).

Acquired resistance to BRAF inhibitors is thought to be due to feedback reactivation of MAPK signaling and mutations in MAPK pathway genes, such as MAP2K1, GNAS, KRAS, and MEK, among others [44]. In ctDNA analysis of specimens from the BEACON trial, the most common alterations noted with both doublet and triplet therapy were KRAS and NRAS mutations and MET amplification. The doublet arm demonstrated a greater rate of MAP2K1 mutations at 16.1% relative to only 3.6% in the triplet arm [59]. Ongoing trials to overcome acquired resistance to BRAF inhibition, in addition to combination with ERK inhibition as noted above, include inhibition of SHP2, a phosphatase upstream in the MAPK pathway, and inhibition of MUC1-C, a transmembrane oncogenic signaling protein [60,61].

### 3.4. KRAS

Kirsten rat sarcoma (KRAS) is an oncogene encoding a guanosine triphosphatase signal transduction protein in the MAPK pathway [62] (Figure 1). KRAS mutations are a poor prognostic indicator and are associated with right-sided primary tumors and anti-EGFR treatment resistance [62]. KRAS mutations are associated with inferior outcomes among patients with microsatellite stable (MSS) tumors; however, this effect is not seen in MSI-H CRC tumors [63]. Mutations in KRAS are seen in approximately 40% of CRC patients, with KRAS G12C, a mutation involving substitution of glycine at codon 12 for cysteine, occurring in about 3% of CRC patients. KRAS G12C leads to a constitutively active protein conformation that promotes tumorigenesis [62,64]. While KRAS G12C inhibitors have shown benefit in non-small cell lung cancer, there has been limited efficacy as monotherapy in mCRC trials. In CodeBreaK100, a phase I trial of 129 patients with KRAS G12C-mutated solid tumors treated with KRAS G12C inhibitor sotorasib, of the 42 patients with CRC only 3 patients (7.1%) had a disease response [65]. Similar results were shown in the phase II CodeBreaK100 study. Of the 62 patients with KRAS G12C mutant CRC, only 6 patients (9.7%) had a disease response with sotorasib monotherapy [66] (Table 1).

Despite lackluster KRAS G12C inhibitor monotherapy efficacy in CRC, recent studies have shown encouraging results with combined targeted therapy approaches (Figure 2). Through preclinical investigation of a KRAS G12C mutant CRC cell line, upregulation of EGFR signaling was found to be a potential mechanism of resistance to KRAS G12C inhibition [67]. In KRYSTAL-1, a phase I/II trial of KRAS G12C mutant mCRC patients, treatment with monotherapy KRAS G12C inhibitor adagrasib was compared to adagrasib plus cetuximab. Adagrasib monotherapy demonstrated a 19% response rate, compared to a 46% response rate in the combination group [68]. To further investigate this favorable response, investigators have initiated KRYSTAL-10, a phase III trial of adagrasib plus cetuximab versus chemotherapy in patients with previously treated KRAS G12C mutant mCRC [69]. In addition, sotorasib is also being investigated in combination with panitumumab in patients with previously treated KRAS G12C CRC in the ongoing phase III CodeBreak 300 trial [70]. Other ongoing trials investigating novel targeted therapies in KRAS mutant CRC include trials of KRAS G12D inhibitors and pan-KRAS inhibitors, among others [71,72,73,74] (Table 2).

Mechanisms of acquired resistance to KRAS G12C inhibitors include MAPK pathway mutations, transformation from adenocarcinoma to squamous histology, alternative KRAS alterations, and a high degree of KRAS G12C amplification [75]. As with efforts to overcome BRAF-inhibition-acquired resistance, combination with SHP2 inhibition is being evaluated as one method to overcome acquired resistance to KRAS G12C inhibition. The ongoing KRYSTAL 2 trial is evaluating adagrasib plus SHP2 inhibitor (TNO155) in advanced solid tumors, including CRC, with KRAS G12C mutations [76]. Other proposed mechanisms of resistance to KRAS G12C inhibition, such as PI3K/AKT/mTOR pathway and associated upstream receptor tyrosine kinase mutations, are being targeted with the addition of mTOR inhibitor, everolimus, and IGF1 receptor inhibitor, linsitinib [77]. Combination therapy with CDK4/6 inhibitors and immunotherapy to overcome KRAS G12C inhibitor resistance is also under pre-clinical investigation [78,79].

### 3.5. MSI/MMR

MSI-H, dMMR, and elevated tumor mutation burden (TMB) are three biomarkers associated with immunotherapy efficacy in CRC. Errors in DNA replication are repaired by the mismatch repair (MMR) system, composed of a series of protein complexes involved in DNA replication error recognition, excision, and repair [80]. Mutations in genes encoding MMR proteins can lead to a malfunction, or deficiency, in the ability of the MMR system to function properly, known as dMMR. Patients with dMMR tumors can accumulate DNA mutations at high rates, particularly in DNA segments known as microsatellites, short DNA sequences that are repeated in tandem [81]. Accumulation of microsatellite mutations leads to variability in microsatellite length and sequence, both of which can be detected and quantified as MSI-H. As such, MSI-H is often seen as a result of, and in concordance with, dMMR [82]. TMB refers to the quantity of mutations noted in a tumor sample that, when elevated, is associated with increased neoantigen production and increased T cell infiltration into the tumor microenvironment, which results in increased response to immunotherapy [83]. While dMMR is seen in 10–20% of CRCs, it is only noted in approximately 3.5% of metastatic CRCs [84]. Similarly, MSI-H is seen in 10–15% of CRCs and 2.7% of mCRCs [85]. MSI-H CRC has been associated with primary lesions in the proximal colon and an improved prognosis compared to MSS CRC [86].

Currently approved immunotherapy agents for mCRC include monotherapy with anti-PD-1 antibodies pembrolizumab or nivolumab, and combination nivolumab plus anti-CTLA-4 antibody, ipilimumab, in dMMR or MSI-H mCRC (Figure 1). Nivolumab demonstrated encouraging results in previously treated dMMR/MSI-H mCRC in the phase II CheckMate 142 trial. In this study, nivolumab was administered to 74 patients with previously treated dMMR/MSI-H mCRC. The overall response rate (ORR) was 31.1% (95% CI 20.8–42.9%) and the most common adverse events included fatigue and gastrointestinal side effects. Based on results from this study, nivolumab obtained FDA approval for previously treated MSI-H/dMMR mCRC in July 2017 [87,88] (Table 1). In contrast to pembrolizumab, first-line nivolumab monotherapy has not been assessed. Combination nivolumab plus ipilimumab was evaluated in previously treated MSI-H/dMMR mCRC in a cohort of patients in CheckMate 142. Of the 119 patients treated with combination nivolumab plus ipilimumab, the ORR was 55% (95% CI 45.2–63.8%) with a PFS of 71% at 12 months. Grade 3 or greater adverse events occurred in 32% of patients [89] (Table 1). With these improved outcomes relative to nivolumab monotherapy, in July 2018, the FDA approved nivolumab plus ipilimumab for previously treated MSI-H/dMMR mCRC [90]. Although there is higher efficacy with combination nivolumab plus ipilimumab compared to nivolumab monotherapy, combination therapy results in a higher rate of immunotherapy-related adverse events with multiorgan involvement [91].

Pembrolizumab was the first FDA-approved immunotherapy agent for previously treated MSI-H/dMMR mCRC in May 2017 [92,93,94]. This was a tissue-agnostic approval for all MSI-H/dMMR solid tumors and the first tissue-agnostic approval for any cancer drug. This groundbreaking approval led to further tumor-specific and first-line treatment trials. Pembrolizumab efficacy in first-line treatment of MSI-H mCRC was evaluated in KEYNOTE-177. In this phase III trial, 307 patients with previously untreated MSI-H CRC were randomized to treatment with pembrolizumab versus chemotherapy. The ORR was 43.8% in the pembrolizumab group relative to 33.1% in the chemotherapy group. Median PFS was 16.5 months with pembrolizumab versus 8.2 months with chemotherapy with a hazard ratio of 0.60 (95% CI 0.45–0.80). Grade 3 or greater treatment-related adverse events occurred in 22% of patients in the pembrolizumab group, with gastrointestinal side effects and fatigue being the most common. This was a favorable adverse event profile compared to chemotherapy, which demonstrated a grade 3 or greater treatment-related adverse event rate of 66% [95]. Based on these encouraging results, pembrolizumab was approved as a first-line treatment option for MSI-H mCRC patients (Figure 2).

There has been recent progress with immunotherapy in non-metastatic CRC. The NICHE trial evaluating neoadjuvant nivolumab plus ipilimumab in locally advanced dMMR and MMR-stable CRC demonstrated a response rate of 100% among dMMR patients and a pathologic complete response in 69% (95% CI 53–85%) of patients. Grade 3 immunotherapy-related adverse events occurred in 12% of patients [96]. Similarly impressive results were demonstrated in NICHE-2, a trial with a larger cohort of 112 patients with dMMR CRC [97]. PD-1 inhibitor dostarlimab also demonstrated encouraging results in the neoadjuvant setting. In a phase II trial of 12 patients with dMMR locally advanced rectal adenocarcinoma, neoadjuvant dostarlimab resulted in a clinical complete response in 100% of patients and no patients received subsequent chemoradiation or surgery as a result of their complete response by the time of study publication [94]. There were no grade 3 or greater adverse events reported [98]. Immunotherapy is currently being evaluated in the adjuvant setting in the ATOMIC trial, a phase III trial of PD-L1 inhibitor atezolizumab combined with chemotherapy versus chemotherapy alone in patients with dMMR resected stage III CRC [99]. There are numerous ongoing trials with novel immunotherapy agents both in the metastatic setting and in the neoadjuvant/adjuvant settings [100,101,102,103] (Table 2).

Resistance to immunotherapy is multifactorial and in part driven by the tumor microenvironment [104,105]. MSS CRC tumors, which are considered poorly responsive to immunotherapy, display greater levels of tumor-associated macrophages than MSI-H tumors [104]. TCGA analysis across 31 solid tumor types demonstrates that increased Wnt/B-catenin signaling pathway activation has been associated with decreased T cell tumor infiltration which is associated with decreased immunotherapy efficacy [105].

### 3.6. HER2

Human epidermal growth factor receptor 2 (HER2) is a gene encoding a receptor tyrosine kinase that functions through dimerization with other receptor tyrosine kinases and is involved in downstream activation of multiple cellular proliferation pathways, including MAPK and PI3K, among others (Figure 1). HER2 gene amplification and/or overexpression of the HER2 protein can result in increased downstream proliferative signaling. HER2 amplification has been noted in up to 6% of CRC cases [106]. HER2-activating mutations have also been identified in about 3% of mCRCs [107]. Clinically, HER2-positive CRC has been associated with distal colorectal primary lesions and with lung and brain metastases [108]. Regarding associated molecular alterations, HER2 amplification occurs with RAS mutations in 20% of cases [109]. Meanwhile, HER2-mutant CRC is associated with high TMB and MSI-H tumors [107].

Dual HER2 inhibition with anti-HER2 antibody, trastuzumab, and small molecule HER2 and EGFR inhibitor, lapatinib, was investigated in HERACLES, a phase II trial in 33 patients with previously treated HER2 positive mCRC. Results demonstrated an ORR of 31% (95% CI 16–49%). Grade 3 adverse events were noted in 22% of patients, with toxicities including fatigue, skin rash, and hyperbilirubinemia [110] (Table 1). A subset analysis of HER2-amplified mCRC was performed on patients from MyPathway, a phase II basket trial of HER2-amplified advanced solid tumors treated with trastuzumab plus pertuzumab, an anti-HER2 antibody that prevents HER2/HER3 heterodimerization. Among 57 patients with HER2-amplified mCRC treated with trastuzumab and pertuzumab, the ORR was 32% (95% CI 20–45%). Grade 3 or greater adverse events occurred in 37% of patients, the most common of which were abdominal pain and hypokalemia [111]. Despite the encouraging and potentially similar outcomes with trastuzumab plus lapatinib and trastuzumab plus pertuzumab in the HERACLES and MyPathway trials, there were less robust outcomes with trastuzumab plus pertuzumab noted in the TAPUR study, a phase II basket trial of targeted therapies in patients with advanced cancers and genomic alterations. Of the 28 patients with HER2-amplified CRC treated with trastuzumab plus pertuzumab, the ORR was 14% (95% CI 4–33%) [112]. MOUNTAINEER was a phase II trial investigating trastuzumab plus tucatinib, a small molecule selective HER2 inhibitor, in patients with previously treated HER2-positive mCRC. Of the 84 patients evaluated, the ORR was 38% (95% CI 28–49%). Common adverse events included gastrointestinal side effects, fatigue, abdominal pain, infusion reactions, and fevers [113]. Resistance to HER2 therapy is associated with increased activation or mutation of downstream signaling proteins, such as PI3K or RAS [108]. In addition, HER2 mutations and HER3 overexpression have also been associated with HER2 therapy resistance [114].

In addition to dual HER2 inhibition, antibody–drug conjugates (ADCs) have demonstrated efficacy in this subset of mCRC patients (Figure 2). Fam-trastuzumab deruxtecan is an ADC composed of trastuzumab linked to topoisomerase I inhibitor deruxtecan. In the phase II trial DESTINY-CRC01, trastuzumab deruxtecan was evaluated in 78 patients with previously treated HER2-overexpressing mCRC. The ORR was 45.3% (95% CI 31.6–59.6%) and responses were seen among patients who had previously received and progressed on anti-HER2 therapies. While there was a robust response rate of 57.5% among patients with HER2 3+ IHC disease, only one of thirteen patients (7.6%) with HER2 2+ IHC disease responded and no patients in the HER2 1+ IHC group responded. Grade 3 or greater adverse events that occurred most commonly were neutropenia in 22% of patients and anemia in 14% of patients. Five patients (6%) had interstitial lung disease or pneumonitis, two of whom died and were classified as drug-related deaths [115]. Ongoing trials are investigating established HER2 therapies in earlier lines of treatment, as with first-line tucatinib plus trastuzumab plus chemotherapy in MOUNTAINEER-03, along with novel HER2-targeted therapy agents that have the potential to become standard-of-care options in the future [116,117,118,119,120,121,122] (Table 2).

### 3.7. RET

RET, an abbreviation for “rearranged during transfection”, is a gene that encodes a receptor tyrosine kinase (Figure 1). Chromosomal rearrangement can result in a fusion RET kinase that is constitutively activated, leading to downstream MAPK pathway activation that promotes tumorigenesis [123]. RET fusions have been identified in 0.2% of CRC [124]. RET fusion kinase has been associated with young patients without a prior smoking history in NSCLC [125]. However, RET fusions in mCRC have been associated with older age and also with MSI-H disease [126]. A RET inhibitor, selpercatinib, was FDA approved in September 2022 for patients with RET gene fusions and previously treated advanced or metastatic solid tumors [127] (Figure 2). This tissue-agnostic approval was based on the Libretto-001 trial. In this phase I/II basket trial, 45 patients with RET-fusion-positive solid tumors were treated with selpercatinib with an ORR of 43.9% (95% CI 28.5–60.3%). Grade 3 or greater treatment-related adverse events included hypertension (22%), ALT elevation (16%) and AST elevation (13%) [128] (Table 1). Regarding RET-fusion-positive CRC patients, 22% of patients in this study with CRC had a response rate of 20.0%. Despite the lower response rate, the median duration of response was 9.2 months.

### 3.8. NTRK

Neurotrophic tyrosine receptor kinase (NTRK) genes encode tropomyosin receptor kinase (Trk) proteins involved in neuronal development and downstream MAPK and PI3K pathway signaling (Figure 1). Chromosomal translocations involving NTRK genes can result in constitutively active Trk proteins, promoting oncogenesis [129]. NTRK fusions have been identified in approximately 0.7% of CRCs [130]. Clinically, CRCs with NTRK alterations have been associated with a poor prognosis and right-sided disease [131]. Among NTRK-altered CRC patients, there is an elevated median TMB relative to the general CRC population [130]. In addition, approximately 75% of mCRC patients with NTRK alterations are MSI-H and almost 50% have MMR mutations [130]. While these findings suggest possible benefits of immunotherapy in patients with NTRK alterations, the sparsity of patients in this subgroup has limited investigation to individual case reports which have demonstrated mixed responses [132].

In November 2018, the FDA approved the NTRK inhibitor Larotrectinib for NTRK-fusion-positive solid tumors based on the LOXO-TRK-14001, SCOUT, and NAVIGATE trials (Figure 2). Among the 55 patients to receive larotrectinib, there was a 75% response rate (95% CI 61–85%). There were four patients with colon cancer among all three trials. Although CRC subgroup analysis was not performed, all solid tumor subgroups demonstrated responses [133] (Table 1). Common adverse events included fatigue, gastrointestinal side effects, and transaminitis [134]. Subsequently, in August 2019, the FDA approved a second NTRK inhibitor, entrectinib, for patients with NTRK-fusion-positive solid tumors based on the ALKA, STARTRK-1, and STARTRK-2 trials (Figure 1). Through these trials, 54 patients treated with entrectinib demonstrated a response rate of 57% (95% CI 43–71%). Similar to data for larotrectinib, there were four patients with CRC among all three trials and subgroup analysis was not performed [135]. Common adverse events of entrectinib, similar to larotrectinib, included fatigue and gastrointestinal side effects, as well as cognitive and visual disorders [136].

**Table 1:** Systemic therapy options in colorectal cancer. CAPOX = capecitabine plus oxaliplatin. FOLFOX = fluorouracil plus oxaliplatin. FOLFIRI = fluorouracil plus irinotecan. FOLFOXIRI = fluorouracil plus oxaliplatin plus irinotecan. MSI-H = high microsatellite instability. dMMR = mismatch repair deficient.

Therapeutic Class	Therapeutic Agent	Molecular Target	Reference
Targeted Therapy	Bevacizumab	VEGF	[24,25]
	Regorafenib	Multiple kinases: VEGFR1-3, PDGFR, RAF, FGFR1-2, among others	[29]
	Fruquintinib	VEGFR1-3 kinases	[31]
	Cetuximab, Panitumumab	EGFR	[36,38]
	Encorafenib	BRAF^V600E^	[47]
	Adagrasib, Sotorasib	KRAS^G12C^	[68,70]
	Trastuzumab	HER2 extracellular domain (ECD) IV; inhibits ligand-independent HER2 signaling	[110]
	Pertuzumab	HER2 ECD II; inhibits ligand-dependent HER2 signaling	[111]
	Trastuzumab Deruxtecan	HER2-directed antibody-drug (chemotherapy) conjugate (ADC)	[115]
	Lapatinib	EGFR and HER2 kinases	[110]
	Tucatinib	HER2 kinase	[113]
	Selpercatinib	RET	[127]
	Larotrectinib, Entrectinib	NTRK	[133,135]
Immunotherapy (MSI-H/dMMR)	Pembrolizumab, Nivolumab	PD-1	[87,92]
	Ipilimumab	CTLA-4	[89]
Chemotherapy	CAPOX, FOLFOX, FOLFIRI, FOLFOXIRI		[24,26,36,38]

**Table 2:** Select ongoing trials of targeted therapy and immunotherapy in CRC.

Molecular Alteration Targeted	ClinicalTrials.gov Identifier and Trial Name (If Applicable)	Trial Phase	Experimental Agent(s)	Disease Stage(s)	Line of Treatment	Estimated Study Completion Date
BRAF	NCT05510895 (NeoBRAF)	II	Encorafenib plus Binimetinib plus Cetuximab	Resectable T3-4,N−/+,M0	Neoadjuvant and Adjuvant	January 2025
	NCT05743036	I/II	ZN-c3 plus Encorafenib plus Cetuximab	Stage IV	2L+	September 2026
	NCT05308446	II	Cetuximab plus Encorafenib plus Nivolumab	Stage IV	2L+	August 2024
	NCT05127759	II	HLX208	Stage IV	2L+	February 2025
KRAS	NCT05593328	II	Onvansertib plus FOLFIRI plus Bevacizumab	Stage IV	2L+	April 2026
	NCT05631574	I	BMF-219	Advanced	2L+	October 2026
	NCT04117087	I	KRAS peptide vaccine plus Nivolumab plus Ipilimumab	Stage IV	3L+	December 2024
	NCT05379985	I	RMC-6236	Advanced	2L+	December 2025
	NCT05737706	I/II	MRTX1133	Advanced	2L+	August 2026
MSI-H/dMMR	NCT04895722	II	Pembrolizumab plus Quavonlimab or Favezelimab or Vibostolimab or MK-4830	Stage IV	1L+	October 2025
	NCT05652894	III	HX008	Stage IV	1L	October 2028
	NCT04988191	I/II	Toripalimab plus Bevacizumab plus Irinotecan	Resectable T3-4 rectal or T1-2 rectal within 12cm of anal verge or T4a-b colon	Neoadjuvant and Adjuvant	December 2023
	NCT05371197	II	Envafolimab	Resectable T3-4,N1-2, M0	Neoadjuvant	December 2024
HER2	NCT05578287 (DETECT)	II	Disitamab Vedotin, Tislelizumab, Capecitabine, and Celecoxib	Stage IV	2L+	December 2025
	NCT05350917	II	Disitamab Vedotin, Tislelizumab, and Pyrotinib Maleate	Advanced	2L+	June 2026
	NCT05785325	II	RC48-ADC plus Bevacizumab	Stage IV	2L+	December 2024
	NCT03929666	II	Zanidatamab plus chemotherapy	Advanced	1L	April 2024
	NCT05673512	II/III	IAH0968 plus CAPEOX	Advanced	1L	March 2026
	NCT05253651 (MOUNTAINEER-03)	III	Tucatinib plus trastuzumab plus mFOLFOX6	Stage IV	1L	April 2028
	NCT05356897 (3T Study)	II	Tucatinib plus Trastuzumab plus TAS-102	Stage IV	2L+	May 2029

### 3.9. Other Targeted Therapies

Ongoing studies in CRC are investigating additional targeted therapies that have demonstrated efficacy in other malignancies. PIK3CA is a gene encoding for p110a involved in the PI3K/AKT/mTOR cellular proliferation and survival signaling pathway. Mutations in PIK3CA are known to promote carcinogenesis in CRC and are identified in approximately 20% of CRC patients [137,138]. Alpelisib is a PIK3CA inhibitor currently used in breast cancer treatment [139]. Alpelisib as an adjunctive treatment was studied in a phase II trial of 102 patients with previously treated advanced BRAF-mutant CRC treated with encorafenib plus cetuximab with or without alpelisib. On planned PFS analysis, triplet therapy demonstrated a median PFS of 5.4 months (95% CI 4.1–7.2 months) compared to a median PFS of 4.2 months (95% CI 3.4–5.4 months) with doublet therapy with a hazard ratio of 0.69 (95% CI 0.43–1.11) [140]. Alpelisib monotherapy was also evaluated in a phase IA basket trial of 134 patients with PIK3CA-mutated advanced solid tumors. Among the 35 patients with CRC, while only 2 patients demonstrated response, 10 patients were noted to have stable disease, together yielding a disease control rate of 34.3% (95% CI 19.1–52.2%). The most common adverse events included hyperglycemia, nausea, decreased appetite, and diarrhea [141].

The FGFR inhibitor pemigatinib has been evaluated in FGFR-altered mCRC in the phase II ACCRU-GI-1701 trial. No objective responses were observed among the 12 patients treated with pemigatinib [142]. C-Ros oncogene 1, receptor tyrosine kinase (ROS1) rearrangements, while commonly targeted in lung cancer, are quite rare to even detect on NGS in CRC at a rate of 0.08% among 40,589 patients screened with genomic profiling. However, one index patient with ROS1-rearranged chemotherapy refractory mCRC treated with ROS1 inhibitor crizotinib experienced a sustained partial response of 15 months [143]. Similarly, anaplastic lymphoma kinase (ALK) rearrangements, more commonly targeted in lung cancer, have rarely been noted in CRC. A case report of ALK-rearranged mCRC treated with the ALK inhibitor alectinib demonstrated a partial response for 8 months [144].

## 4. Young-Onset CRC

Young-onset CRC is a subset of CRC occurring in individuals under 50 years of age. Clinically, young-onset CRC has a greater propensity than standard-onset CRC to be associated with left-sided disease, abdominal pain, and rectal bleeding [145]. It is associated with worse outcomes and has distinct molecular alterations compared to average-onset CRC [4,146,147]. Patients with young-onset CRC have a higher prevalence of germline mutations in known oncogenes, such as MUTYH, SMAD4, BRCA1, BRCA2, and PALB2 among others, and therefore patients are recommended to undergo genetic testing/counseling [147]. An estimated 16–35% of young-onset CRC cases have been associated with hereditary cancer syndromes, 34–71% of which are cases of Lynch syndrome [146]. Lynch syndrome is characterized by germline mutations in DNA mismatch repair genes [148]. The second most common hereditary form of CRC is familial adenomatous polyposis, characterized by APC gene mutations, and approximately 20% of young-onset CRCs have a pattern of familial inheritance without a specific heritable mutation identified [149]. Unfortunately, only a minority of patients currently undergo the recommended genetic counseling, and thus, hereditary CRC is thought to be underdiagnosed even in young-onset CRC [150]. From TCGA analysis, young-onset CRC was noted to have a higher propensity of mutations in the MMR and PI3K pathway genes compared to patients with average-onset CRC, yet with the addition of proteomic analysis, only two MMR proteins, MSH2 and MSH6, were found to have decreased expression in young-onset CRC [151]. Using multi-omics analysis, which integrates genomic, transcriptomic, and proteomic analysis, researchers have identified dysregulated redox status to be a distinct molecular profile in young-onset sporadic MSS CRC via the NRF2-mediated oxidative stress response pathway and CXCL12-CXCR4 signaling, among other mechanisms [152].

## 5. Conclusions and Future Directions

In conclusion, the rapid growth of molecular analysis techniques, targeted therapies, and immunotherapy has brought personalized medicine to the forefront of CRC treatment and improved patient outcomes. With the high burden of CRC globally and specifically the worsening outcomes in early-onset CRC, there is an unmet need for better understanding the molecular mechanisms underlying CRC development, which will facilitate development of novel therapeutic strategies against CRC.

## References

[1] Siegel R.L., Miller K.D., Wagle N.S., Jemal A. (2023). Cancer Statistics, 2023. CA Cancer J. Clin..

[2] Sung H., Ferlay J., Siegel R.L., Laversanne M., Soerjomataram I., Jemal A., Bray F. (2021). Global Cancer Statistics 2020: GLOBOCAN Estimates of Incidence and Mortality Worldwide for 36 Cancers in 185 Countries. CA Cancer J. Clin..

[3] Keum N., Giovannucci E. (2019). Global Burden of Colorectal Cancer: Emerging Trends, Risk Factors and Prevention Strategies. Nat. Rev. Gastroenterol. Hepatol..

[4] Sinicrope F.A. (2022). Increasing Incidence of Early-Onset Colorectal Cancer. N. Engl. J. Med..

[5] Holch J.W., Ricard I., Stintzing S., Modest D.P., Heinemann V. (2017). The Relevance of Primary Tumour Location in Patients with Metastatic Colorectal Cancer: A Meta-Analysis of First-Line Clinical Trials. Eur. J. Cancer.

[6] Kapiteijn E., Liefers G.J., Los L.C., Kranenbarg E.K., Hermans J., Tollenaar R.A., Moriya Y., van de Velde C.J., van Krieken J.H. (2001). Mechanisms of Oncogenesis in Colon versus Rectal Cancer. J. Pathol..

[7] Chakravarty D., Johnson A., Sklar J., Lindeman N.I., Moore K., Ganesan S., Lovly C.M., Perlmutter J., Gray S.W., Hwang J. (2022). Somatic Genomic Testing in Patients with Metastatic or Advanced Cancer: ASCO Provisional Clinical Opinion. J. Clin. Oncol..

[8] (2023). NCCN Clinical Practice Guidelines in Oncology Colon Cancer Version 1.2023.

[9] Malla M., Loree J.M., Kasi P.M., Parikh A.R. (2022). Using Circulating Tumor DNA in Colorectal Cancer: Current and Evolving Practices. J. Clin. Oncol..

[10] Sullivan B.G., Lo A., Yu J., Gonda A., Dehkordi-Vakil F., Dayyani F., Senthil M. (2023). Circulating Tumor DNA Is Unreliable to Detect Somatic Gene Alterations in Gastrointestinal Peritoneal Carcinomatosis. Ann. Surg. Oncol..

[11] Nakamura Y., Taniguchi H., Ikeda M., Bando H., Kato K., Morizane C., Esaki T., Komatsu Y., Kawamoto Y., Takahashi N. (2020). Clinical Utility of Circulating Tumor DNA Sequencing in Advanced Gastrointestinal Cancer: SCRUM-Japan GI-SCREEN and GOZILA Studies. Nat. Med..

[12] Parikh A.R., Van Seventer E.E., Siravegna G., Hartwig A.V., Jaimovich A., He Y., Kanter K., Fish M.G., Fosbenner K.D., Miao B. (2021). Minimal Residual Disease Detection Using a Plasma-Only Circulating Tumor DNA Assay in Patients with Colorectal Cancer. Clin. Cancer Res..

[13] Ten Hoorn S., de Back T.R., Sommeijer D.W., Vermeulen L. (2022). Clinical Value of Consensus Molecular Subtypes in Colorectal Cancer: A Systematic Review and Meta-Analysis. J. Natl. Cancer Inst..

[14] Guinney J., Dienstmann R., Wang X., de Reyniès A., Schlicker A., Soneson C., Marisa L., Roepman P., Nyamundanda G., Angelino P. (2015). The Consensus Molecular Subtypes of Colorectal Cancer. Nat. Med..

[15] Zhang B., Wang J., Wang X., Zhu J., Liu Q., Shi Z., Chambers M.C., Zimmerman L.J., Shaddox K.F., Kim S. (2014). Proteogenomic Characterization of Human Colon and Rectal Cancer. Nature.

[16] Vasaikar S., Huang C., Wang X., Petyuk V.A., Savage S.R., Wen B., Dou Y., Zhang Y., Shi Z., Arshad O.A. (2019). Proteogenomic Analysis of Human Colon Cancer Reveals New Therapeutic Opportunities. Cell.

[17] Song H., Zeng J., Roychoudhury S., Biswas P., Mohapatra B., Ray S., Dowlatshahi K., Wang J., Band V., Talmon G. (2020). Targeting Histone Chaperone FACT Complex Overcomes 5-Fluorouracil Resistance in Colon Cancer. Mol. Cancer Ther..

[18] Bhakat K.K., Ray S. (2022). The FAcilitates Chromatin Transcription (FACT) Complex: Its Roles in DNA Repair and Implications for Cancer Therapy. DNA Repair.

[19] Garcia H., Miecznikowski J.C., Safina A., Commane M., Ruusulehto A., Kilpinen S., Leach R.W., Attwood K., Li Y., Degan S. (2013). Facilitates Chromatin Transcription Complex Is an “Accelerator” of Tumor Transformation and Potential Marker and Target of Aggressive Cancers. Cell Rep..

[20] Gasparian A.V., Burkhart C.A., Purmal A.A., Brodsky L., Pal M., Saranadasa M., Bosykh D.A., Commane M., Guryanova O.A., Pal S. (2011). Curaxins: Anticancer Compounds that Simultaneously Suppress NF-κB and Activate p53 by Targeting FACT. Sci. Transl. Med..

[21] Roelands J., Kuppen P.J.K., Ahmed E.I., Mall R., Masoodi T., Singh P., Monaco G., Raynaud C., de Miranda N.F.C.C., Ferraro L. (2023). An Integrated Tumor, Immune and Microbiome Atlas of Colon Cancer. Nat. Med..

[22] Pelka K., Hofree M., Chen J.H., Sarkizova S., Pirl J.D., Jorgji V., Bejnood A., Dionne D., Ge W.H., Xu K.H. (2021). Spatially Organized Multicellular Immune Hubs in Human Colorectal Cancer. Cell.

[23] Carmeliet P. (2005). VEGF as a Key Mediator of Angiogenesis in Cancer. Oncology.

[24] Hurwitz H., Fehrenbacher L., Novotny W., Cartwright T., Hainsworth J., Heim W., Berlin J., Baron A., Griffing S., Holmgren E. (2004). Bevacizumab plus Irinotecan, Fluorouracil, and Leucovorin for Metastatic Colorectal Cancer. N. Engl. J. Med..

[25] Kabbinavar F.F., Hambleton J., Mass R.D., Hurwitz H.I., Bergsland E., Sarkar S. (2005). Combined Analysis of Efficacy: The Addition of Bevacizumab to Fluorouracil/leucovorin Improves Survival for Patients with Metastatic Colorectal Cancer. J. Clin. Oncol..

[26] Goey K.K.H., Elias S.G., van Tinteren H., Laclé M.M., Willems S.M., Offerhaus G.J.A., de Leng W.W.J., Strengman E., Ten Tije A.J., Creemers G.-J.M. (2017). Maintenance Treatment with Capecitabine and Bevacizumab versus Observation in Metastatic Colorectal Cancer: Updated Results and Molecular Subgroup Analyses of the Phase 3 CAIRO3 Study. Ann. Oncol..

[27] Garcia J., Hurwitz H.I., Sandler A.B., Miles D., Coleman R.L., Deurloo R., Chinot O.L. (2020). Bevacizumab (Avastin^®^) in Cancer Treatment: A Review of 15 Years of Clinical Experience and Future Outlook. Cancer Treat. Rev..

[28] Bennouna J., Sastre J., Arnold D., Österlund P., Greil R., Van Cutsem E., von Moos R., Viéitez J.M., Bouché O., Borg C. (2013). Continuation of Bevacizumab after First Progression in Metastatic Colorectal Cancer (ML18147): A Randomised Phase 3 Trial. Lancet Oncol..

[29] Grothey A., Van Cutsem E., Sobrero A., Siena S., Falcone A., Ychou M., Humblet Y., Bouché O., Mineur L., Barone C. (2013). Regorafenib Monotherapy for Previously Treated Metastatic Colorectal Cancer (CORRECT): An International, Multicentre, Randomised, Placebo-Controlled, Phase 3 Trial. Lancet.

[30] Goel G. (2018). Evolution of Regorafenib from Bench to Bedside in Colorectal Cancer: Is It an Attractive Option or Merely a “Me Too” Drug?. Cancer Manag. Res..

[31] Dasari A., Lonardi S., Garcia-Carbonero R., Elez E., Yoshino T., Sobrero A., Yao J., García-Alfonso P., Kocsis J., Cubillo Gracian A. (2023). Fruquintinib versus Placebo in Patients with Refractory Metastatic Colorectal Cancer (FRESCO-2): An International, Multicentre, Randomised, Double-Blind, Phase 3 Study. Lancet.

[32] Sigismund S., Avanzato D., Lanzetti L. (2018). Emerging Functions of the EGFR in Cancer. Mol. Oncol..

[33] Cann C.G., LaPelusa M.B., Cimino S.K., Eng C. (2023). Molecular and Genetic Targets within Metastatic Colorectal Cancer and Associated Novel Treatment Advancements. Front. Oncol..

[34] Karapetis C.S., Khambata-Ford S., Jonker D.J., O’Callaghan C.J., Tu D., Tebbutt N.C., Simes R.J., Chalchal H., Shapiro J.D., Robitaille S. (2008). K-Ras Mutations and Benefit from Cetuximab in Advanced Colorectal Cancer. N. Engl. J. Med..

[35] Grothey A., Fakih M., Tabernero J. (2021). Management of BRAF-Mutant Metastatic Colorectal Cancer: A Review of Treatment Options and Evidence-Based Guidelines. Ann. Oncol..

[36] Douillard J.-Y., Siena S., Cassidy J., Tabernero J., Burkes R., Barugel M., Humblet Y., Bodoky G., Cunningham D., Jassem J. (2010). Randomized, Phase III Trial of Panitumumab with Infusional Fluorouracil, Leucovorin, and Oxaliplatin (FOLFOX4) versus FOLFOX4 Alone as First-Line Treatment in Patients with Previously Untreated Metastatic Colorectal Cancer: The PRIME Study. J. Clin. Oncol..

[37] Yaeger R., Chatila W.K., Lipsyc M.D., Hechtman J.F., Cercek A., Sanchez-Vega F., Jayakumaran G., Middha S., Zehir A., Donoghue M.T.A. (2018). Clinical Sequencing Defines the Genomic Landscape of Metastatic Colorectal Cancer. Cancer Cell.

[38] Tejpar S., Stintzing S., Ciardiello F., Tabernero J., Van Cutsem E., Beier F., Esser R., Lenz H.-J., Heinemann V. (2017). Prognostic and Predictive Relevance of Primary Tumor Location in Patients with RAS Wild-Type Metastatic Colorectal Cancer: Retrospective Analyses of the CRYSTAL and FIRE-3 Trials. JAMA Oncol..

[39] Siravegna G., Mussolin B., Buscarino M., Corti G., Cassingena A., Crisafulli G., Ponzetti A., Cremolini C., Amatu A., Lauricella C. (2015). Clonal Evolution and Resistance to EGFR Blockade in the Blood of Colorectal Cancer Patients. Nat. Med..

[40] Cremolini C., Rossini D., Dell’Aquila E., Lonardi S., Conca E., Del Re M., Busico A., Pietrantonio F., Danesi R., Aprile G. (2019). Rechallenge for Patients with RAS and BRAF Wild-Type Metastatic Colorectal Cancer with Acquired Resistance to First-Line Cetuximab and Irinotecan: A Phase 2 Single-Arm Clinical Trial. JAMA Oncol..

[41] Sartore-Bianchi A., Pietrantonio F., Lonardi S., Mussolin B., Rua F., Crisafulli G., Bartolini A., Fenocchio E., Amatu A., Manca P. (2022). Circulating Tumor DNA to Guide Rechallenge with Panitumumab in Metastatic Colorectal Cancer: The Phase 2 CHRONOS Trial. Nat. Med..

[42] Parseghian C.M., Sun R., Woods M., Napolitano S., Lee H.M., Alshenaifi J., Willis J., Nunez S., Raghav K.P., Morris V.K. (2023). Resistance Mechanisms to Anti-Epidermal Growth Factor Receptor Therapy in RAS/RAF Wild-Type Colorectal Cancer Vary by Regimen and Line of Therapy. J. Clin. Oncol..

[43] Grassi E., Corbelli J., Papiani G., Barbera M.A., Gazzaneo F., Tamberi S. (2021). Current Therapeutic Strategies in BRAF-Mutant Metastatic Colorectal Cancer. Front. Oncol..

[44] Tabernero J., Ros J., Élez E. (2022). The Evolving Treatment Landscape in BRAF-V600E–Mutated Metastatic Colorectal Cancer. Am. Soc. Clin. Oncol. Educ. Book.

[45] Chu J.E., Johnson B., Kugathasan L., Morris V.K., Raghav K., Swanson L., Lim H.J., Renouf D.J., Gill S., Wolber R. (2020). Population-Based Screening for BRAF V600E in Metastatic Colorectal Cancer Reveals Increased Prevalence and Poor Prognosis. Clin. Cancer Res..

[46] Venderbosch S., Nagtegaal I.D., Maughan T.S., Smith C.G., Cheadle J.P., Fisher D., Kaplan R., Quirke P., Seymour M.T., Richman S.D. (2014). Mismatch Repair Status and BRAF Mutation Status in Metastatic Colorectal Cancer Patients: A Pooled Analysis of the CAIRO, CAIRO2, COIN, and FOCUS Studies. Clin. Cancer Res..

[47] Kopetz S., Grothey A., Yaeger R., Van Cutsem E., Desai J., Yoshino T., Wasan H., Ciardiello F., Loupakis F., Hong Y.S. (2019). Encorafenib, Binimetinib, and Cetuximab in BRAF V600E-Mutated Colorectal Cancer. N. Engl. J. Med..

[48] Tabernero J., Grothey A., Van Cutsem E., Yaeger R., Wasan H., Yoshino T., Desai J., Ciardiello F., Loupakis F., Hong Y.S. (2021). Encorafenib Plus Cetuximab as a New Standard of Care for Previously Treated BRAF V600E-Mutant Metastatic Colorectal Cancer: Updated Survival Results and Subgroup Analyses from the BEACON Study. J. Clin. Oncol..

[49] Study of Encorafenib + Cetuximab Plus or Minus Binimetinib vs. Irinotecan/Cetuximab or Infusional 5-Fluorouracil (5-FU)/Folinic Acid (FA)/Irinotecan (FOLFIRI)/Cetuximab with a Safety Lead-in of Encorafenib + Binimetinib + Cetuximab in Patients with BRAF V600E-Mutant Metastatic Colorectal Cancer—Full Text View—ClinicalTrials.gov. https://clinicaltrials.gov/ct2/show/NCT02928224.

[50] Kopetz S., Grothey A., Yaeger R., Ciardiello F., Desai J., Kim T.W., Maughan T., Van Cutsem E., Wasan H.S., Yoshino T. (2021). BREAKWATER: Randomized Phase 3 Study of Encorafenib (enco) + Cetuximab (cetux) ± Chemotherapy for First-Line (1L) Treatment (tx) of BRAF V600E-Mutant (BRAFV600E) Metastatic Colorectal Cancer (mCRC). J. Clin. Oncol..

[51] Kopetz S. (2023). BREAKWATER Safety Lead-in (SLI): Encorafenib (E) + Cetuximab (C) + Chemotherapy for BRAFV600E Metastatic Colorectal Cancer (mCRC). J. Clin. Oncol..

[52] Testing the Addition of Nivolumab to Standard Treatment for Patients with Metastatic or Unresectable Colorectal Cancer that Have a BRAF Mutation. https://clinicaltrials.gov/ct2/show/NCT05308446?term=BRAF&recrs=ab&cond=Colorectal+Cancer&draw=2&rank=31.

[53] Encorafenib, Cetuximab, and Nivolumab in Treating Patients with Microsatellite Stable, BRAFV600E Mutated Unresectable or Metastatic Colorectal Cancer. https://clinicaltrials.gov/ct2/show/NCT04017650?term=NCT04017650&draw=2&rank=1.

[54] Tolerability and Safety of Vemurafenib, Cetuximab Combined with Camrelizumab for BRAF V600E-Mutated/MSS Metastatic Colorectal Cancer. https://clinicaltrials.gov/ct2/show/NCT05019534?term=NCT05019534&draw=2&rank=1.

[55] A Study of Encorafenib Plus Cetuximab Taken Together with Pembrolizumab Compared to Pembrolizumab Alone in People with Previously Untreated Metastatic Colorectal Cancer—Full Text View—ClinicalTrials.gov. https://clinicaltrials.gov/ct2/show/NCT05217446?term=NCT05217446&draw=2&rank=1.

[56] Sullivan R.J., Infante J.R., Janku F., Wong D.J.L., Sosman J.A., Keedy V., Patel M.R., Shapiro G.I., Mier J.W., Tolcher A.W. (2018). First-in-Class ERK1/2 Inhibitor Ulixertinib (BVD-523) in Patients with MAPK Mutant Advanced Solid Tumors: Results of a Phase I Dose-Escalation and Expansion Study. Cancer Discov..

[57] Ciombor K.K., Strickler J.H., Bekaii-Saab T.S., Yaeger R. (2022). BRAF-Mutated Advanced Colorectal Cancer: A Rapidly Changing Therapeutic Landscape. J. Clin. Oncol..

[58] Neoadjuvant Encorafenib, Binimetinib and Cetuximab for Patients with BRAF V600E Mutated/pMMR Localized Colorectal Cancer—Full Text View—ClinicalTrials.gov. https://clinicaltrials.gov/ct2/show/NCT05510895?term=BRAF&recrs=ab&cond=Colorectal+Cancer&draw=2&rank=17.

[59] Kopetz S., Murphy D.A., Pu J., Yaeger R., Ciardiello F., Desai J., Van Cutsem E., Wasan H.S., Yoshino T., Alkuzweny B. (2022). Genomic Mechanisms of Acquired Resistance of Patients (pts) with BRAF V600E-Mutant (mt) Metastatic Colorectal Cancer (mCRC) Treated in the BEACON Study. Ann. Oncol..

[60] Liu C., Lu H., Wang H., Loo A., Zhang X., Yang G., Kowal C., Delach S., Wang Y., Goldoni S. (2021). Combinations with Allosteric SHP2 Inhibitor TNO155 to Block Receptor Tyrosine Kinase Signaling. Clin. Cancer Res..

[61] Morimoto Y., Yamashita N., Hirose H., Fushimi A., Haratake N., Daimon T., Bhattacharya A., Ahmad R., Suzuki Y., Takahashi H. (2023). MUC1-C Is Necessary for SHP2 Activation and BRAF Inhibitor Resistance in BRAF(V600E) Mutant Colorectal Cancer. Cancer Lett..

[62] Zhu G., Pei L., Xia H., Tang Q., Bi F. (2021). Role of Oncogenic KRAS in the Prognosis, Diagnosis and Treatment of Colorectal Cancer. Mol. Cancer.

[63] Taieb J., Le Malicot K., Shi Q., Penault-Llorca F., Bouché O., Tabernero J., Mini E., Goldberg R.M., Folprecht G., Luc Van Laethem J. (2017). Prognostic Value of BRAF and KRAS Mutations in MSI and MSS Stage III Colon Cancer. J. Natl. Cancer Inst..

[64] Ji J., Wang C., Fakih M. (2022). Targeting KRASG12C-Mutated Advanced Colorectal Cancer: Research and Clinical Developments. OncoTargets Ther..

[65] Hong D.S., Fakih M.G., Strickler J.H., Desai J., Durm G.A., Shapiro G.I., Falchook G.S., Price T.J., Sacher A., Denlinger C.S. (2020). KRASG12C Inhibition with Sotorasib in Advanced Solid Tumors. N. Engl. J. Med..

[66] Fakih M.G., Kopetz S., Kuboki Y., Kim T.W., Munster P.N., Krauss J.C., Falchook G.S., Han S.-W., Heinemann V., Muro K. (2022). Sotorasib for Previously Treated Colorectal Cancers with KRASG12C Mutation (CodeBreaK100): A Prespecified Analysis of a Single-Arm, Phase 2 Trial. Lancet Oncol..

[67] Amodio V., Yaeger R., Arcella P., Cancelliere C., Lamba S., Lorenzato A., Arena S., Montone M., Mussolin B., Bian Y. (2020). EGFR Blockade Reverts Resistance to KRASG12C Inhibition in Colorectal Cancer. Cancer Discov..

[68] Yaeger R., Weiss J., Pelster M.S., Spira A.I., Barve M., Ou S.-H.I., Leal T.A., Bekaii-Saab T.S., Paweletz C.P., Heavey G.A. (2023). Adagrasib with or without Cetuximab in Colorectal Cancer with Mutated KRAS G12C. N. Engl. J. Med..

[69] Phase 3 Study of MRTX849 with Cetuximab vs Chemotherapy in Patients with Advanced Colorectal Cancer with KRAS G12C Mutation (KRYSTAL-10). https://clinicaltrials.gov/ct2/show/NCT04793958?term=NCT04793958&draw=2&rank=1.

[70] Sotorasib and Panitumumab Versus Investigator’s Choice for Participants with Kirsten Rat Sarcoma (KRAS) p.G12C Mutation—Full Text View—ClinicalTrials.gov. https://clinicaltrials.gov/ct2/show/NCT05198934?term=NCT05198934&draw=2&rank=1.

[71] Study of Onvansertib in Combination with FOLFIRI and Bevacizumab Versus FOLFIRI and Bevacizumab for Second Line Treatment of Metastatic Colorectal Cancer in Participants with a Kirsten Rat Sarcoma Virus Gene (KRAS) or Neuroblastoma-RAS (NRAS) Mutation. https://clinicaltrials.gov/ct2/show/NCT05593328?term=KRAS&recrs=ab&cond=Colorectal+Cancer&draw=2&rank=3.

[72] Study of Covalent Menin Inhibitor BMF-219 in Adult Patients with KRAS Driven Non-Small Cell Lung Cancer, Pancreatic Cancer, and Colorectal Cancer. https://clinicaltrials.gov/ct2/show/NCT05631574?term=KRAS&recrs=ab&cond=Colorectal+Cancer&draw=2&rank=8.

[73] Evaluation of RMC-6236 in Subjects with Advanced Solid Tumors Harboring Specific Mutations in KRAS. https://clinicaltrials.gov/ct2/show/NCT05379985?term=KRAS&recrs=ab&cond=Colorectal+Cancer&draw=2&rank=18.

[74] Study of MRTX1133 in Patients with Advanced Solid Tumors Harboring a KRAS G12D Mutation. https://clinicaltrials.gov/ct2/show/NCT05737706?term=KRAS&recrs=ab&cond=Colorectal+Cancer&draw=2&rank=31.

[75] Lietman C.D., Johnson M.L., McCormick F., Lindsay C.R. (2022). More to the RAS Story: KRASG12C Inhibition, Resistance Mechanisms, and Moving Beyond KRASG12C. Am. Soc. Clin. Oncol. Educ. Book.

[76] Sabari J.K., Park H., Tolcher A.W., Ou S.-H.I., Garon E.B., George B., Janne P.A., Moody S.E., Tan E.Y., Sen S.K. (2021). KRYSTAL-2: A Phase I/II Trial of Adagrasib (MRTX849) in Combination with TNO155 in Patients with Advanced Solid Tumors with KRAS G12C Mutation. J. Clin. Oncol..

[77] Molina-Arcas M., Moore C., Rana S., van Maldegem F., Mugarza E., Romero-Clavijo P., Herbert E., Horswell S., Li L.-S., Janes M.R. (2019). Development of Combination Therapies to Maximize the Impact of KRAS-G12C Inhibitors in Lung Cancer. Sci. Transl. Med..

[78] Lou K., Steri V., Ge A.Y., Hwang Y.C., Yogodzinski C.H., Shkedi A.R., Choi A.L.M., Mitchell D.C., Swaney D.L., Hann B. (2019). KRASG12C Inhibition Produces a Driver-Limited State Revealing Collateral Dependencies. Sci. Signal..

[79] Canon J., Rex K., Saiki A.Y., Mohr C., Cooke K., Bagal D., Gaida K., Holt T., Knutson C.G., Koppada N. (2019). The Clinical KRAS(G12C) Inhibitor AMG 510 Drives Anti-Tumour Immunity. Nature.

[80] Fishel R. (2015). Mismatch Repair. J. Biol. Chem..

[81] De’Angelis G.L., Bottarelli L., Azzoni C., De’Angelis N., Leandro G., Di Mario F., Gaiani F., Negri F. (2018). Microsatellite Instability in Colorectal Cancer. Acta Biomed..

[82] Jin Z., Sinicrope F.A. (2022). Mismatch Repair-Deficient Colorectal Cancer: Building on Checkpoint Blockade. J. Clin. Oncol..

[83] Jardim D.L., Goodman A., de Melo Gagliato D., Kurzrock R. (2021). The Challenges of Tumor Mutational Burden as an Immunotherapy Biomarker. Cancer Cell.

[84] Koopman M., Kortman G.A.M., Mekenkamp L., Ligtenberg M.J.L., Hoogerbrugge N., Antonini N.F., Punt C.J.A., van Krieken J.H.J.M. (2009). Deficient Mismatch Repair System in Patients with Sporadic Advanced Colorectal Cancer. Br. J. Cancer.

[85] Heinemann V., Kraemer N., Buchner H., Fischer von Weikersthal L., Decker T., Kiani A., Vehling-Kaiser U., Al-Batran S.-E., Heintges T., Lerchenmuller C.A. (2018). Somatic DNA Mutations, Tumor Mutational Burden (TMB), and MSI Status: Association with Efficacy in Patients (pts) with Metastatic Colorectal Cancer (mCRC) of FIRE-3 (AIO KRK-0306). J. Clin. Oncol..

[86] Boland C.R., Goel A. (2010). Microsatellite Instability in Colorectal Cancer. Gastroenterology.

[87] Overman M.J., McDermott R., Leach J.L., Lonardi S., Lenz H.-J., Morse M.A., Desai J., Hill A., Axelson M., Moss R.A. (2017). Nivolumab in Patients with Metastatic DNA Mismatch Repair-Deficient or Microsatellite Instability-High Colorectal Cancer (CheckMate 142): An Open-Label, Multicentre, Phase 2 Study. Lancet Oncol..

[88] Center for Drug Evaluation Research FDA Grants Nivolumab Accelerated Approval for MSI-H or dMMR Colorectal Cancer. https://www.fda.gov/drugs/resources-information-approved-drugs/fda-grants-nivolumab-accelerated-approval-msi-h-or-dmmr-colorectal-cancer.

[89] Overman M.J., Lonardi S., Wong K.Y.M., Lenz H.-J., Gelsomino F., Aglietta M., Morse M.A., Van Cutsem E., McDermott R., Hill A. (2018). Durable Clinical Benefit with Nivolumab Plus Ipilimumab in DNA Mismatch Repair-Deficient/Microsatellite Instability-High Metastatic Colorectal Cancer. J. Clin. Oncol..

[90] Center for Drug Evaluation Research FDA Grants Accelerated Approval to Ipilimumab for MSI-H or dMMR Metastatic Colorectal Cancer. https://www.fda.gov/drugs/resources-information-approved-drugs/fda-grants-accelerated-approval-ipilimumab-msi-h-or-dmmr-metastatic-colorectal-cancer.

[91] Wang D.Y., Salem J.-E., Cohen J.V., Chandra S., Menzer C., Ye F., Zhao S., Das S., Beckermann K.E., Ha L. (2018). Fatal Toxic Effects Associated with Immune Checkpoint Inhibitors: A Systematic Review and Meta-Analysis. JAMA Oncol..

[92] Office of the Commissioner FDA Approves First Cancer Treatment for Any Solid Tumor with a Specific Genetic Feature. https://www.fda.gov/news-events/press-announcements/fda-approves-first-cancer-treatment-any-solid-tumor-specific-genetic-feature.

[93] Study of MK-3475 in Patients with Microsatellite Unstable (MSI) Tumors (Cohorts A, B and C). https://clinicaltrials.gov/ct2/show/NCT01876511.

[94] Le D.T., Uram J.N., Wang H., Bartlett B.R., Kemberling H., Eyring A.D., Skora A.D., Luber B.S., Azad N.S., Laheru D. (2015). PD-1 Blockade in Tumors with Mismatch-Repair Deficiency. N. Engl. J. Med..

[95] André T., Shiu K.-K., Kim T.W., Jensen B.V., Jensen L.H., Punt C., Smith D., Garcia-Carbonero R., Benavides M., Gibbs P. (2020). Pembrolizumab in Microsatellite-Instability-High Advanced Colorectal Cancer. N. Engl. J. Med..

[96] Verschoor Y.L., van den Berg J., Beets G., Sikorska K., Aalbers A., van Lent A., Grootscholten C., Huibregtse I., Marsman H., Oosterling S. (2022). Neoadjuvant Nivolumab, Ipilimumab, and Celecoxib in MMR-Proficient and MMR-Deficient Colon Cancers: Final Clinical Analysis of the NICHE Study. J. Clin. Oncol..

[97] Chalabi M., Verschoor Y.L., Van Den Berg J., Sikorska K., Beets G., Lent A.V., Grootscholten M.C., Aalbers A., Buller N., Marsman H. (2022). Neoadjuvant Immune Checkpoint Inhibition in Locally Advanced MMR-Deficient Colon Cancer: The NICHE-2 Study. Ann. Oncol..

[98] Cercek A., Lumish M., Sinopoli J., Weiss J., Shia J., Lamendola-Essel M., El Dika I.H., Segal N., Shcherba M., Sugarman R. (2022). PD-1 Blockade in Mismatch Repair-Deficient, Locally Advanced Rectal Cancer. N. Engl. J. Med..

[99] Sinicrope F.A., Ou F.-S., Zemla T., Nixon A.B., Mody K., Levasseur A., Dueck A.C., Dhanarajan A.R., Lieu C.H., Cohen D.J. (2019). Randomized Trial of Standard Chemotherapy Alone or Combined with Atezolizumab as Adjuvant Therapy for Patients with Stage III Colon Cancer and Deficient Mismatch Repair (ATOMIC, Alliance A021502). J. Clin. Oncol..

[100] Evaluation of Co-Formulated Pembrolizumab/Quavonlimab (MK-1308A) Versus Other Treatments in Participants with Microsatellite Instability-High (MSI-H) or Mismatch Repair Deficient (dMMR) Stage IV Colorectal Cancer (CRC) (MK-1308A-008). https://clinicaltrials.gov/ct2/show/NCT04895722?term=dmmr&recrs=ab&cond=Colorectal+Cancer&draw=2&rank=7.

[101] A Study of HX008 Compared to Chemotherapy in the First-Line Treatment of Subjects with MSI-H/dMMR Metastatic Colorectal Cancer. https://clinicaltrials.gov/ct2/show/NCT05652894?term=dmmr&recrs=ab&cond=Colorectal+Cancer&draw=2&rank=8.

[102] Toripalimab Plus Bevacizumab and Chemotherapy as Neoadjuvant Therapy in Advanced MSI-H or dMMR Colorectal Cancer. https://clinicaltrials.gov/ct2/show/NCT04988191?term=dmmr&recrs=ab&cond=Colorectal+Cancer&draw=2&rank=12.

[103] Envafolimab as Neoadjuvant Immunotherapy in Resectable Locally Advanced dMMR/MSI-H Colorectal Cancer. https://clinicaltrials.gov/ct2/show/NCT05371197?term=dmmr&recrs=ab&cond=Colorectal+Cancer&draw=2&rank=14.

[104] Sahin I.H., Ciombor K.K., Diaz L.A., Yu J., Kim R. (2022). Immunotherapy for Microsatellite Stable Colorectal Cancers: Challenges and Novel Therapeutic Avenues. Am. Soc. Clin. Oncol. Educ. Book.

[105] Luke J.J., Bao R., Sweis R.F., Spranger S., Gajewski T.F. (2019). WNT/β-Catenin Pathway Activation Correlates with Immune Exclusion across Human Cancers. Clin. Cancer Res..

[106] Seo A.N., Kwak Y., Kim D.-W., Kang S.-B., Choe G., Kim W.H., Lee H.S. (2014). HER2 Status in Colorectal Cancer: Its Clinical Significance and the Relationship between HER2 Gene Amplification and Expression. PLoS ONE.

[107] Mohamed A.A., Lau D.K., Chau I. (2022). HER2 Targeted Therapy in Colorectal Cancer: New Horizons. Cancer Treat. Rev..

[108] Ahcene Djaballah S., Daniel F., Milani A., Ricagno G., Lonardi S. (2022). HER2 in Colorectal Cancer: The Long and Winding Road From Negative Predictive Factor to Positive Actionable Target. Am. Soc. Clin. Oncol. Educ. Book.

[109] Yoshikawa A., Nakamura Y. (2022). Molecular Basis of HER2-Targeted Therapy for HER2-Positive Colorectal Cancer. Cancers.

[110] Siena S., Sartore-Bianchi A., Trusolino L., Martino C., Bencardino K., Lonardi S., Zagonel V., Leone F., Martinelli E., Ciardiello F. (2016). Final Results of the HERACLES Trial in HER2 Amplified Colorectal Cancer. Ann. Oncol..

[111] Meric-Bernstam F., Hurwitz H., Raghav K.P.S., McWilliams R.R., Fakih M., VanderWalde A., Swanton C., Kurzrock R., Burris H., Sweeney C. (2019). Pertuzumab plus Trastuzumab for HER2-Amplified Metastatic Colorectal Cancer (MyPathway): An Updated Report from a Multicentre, Open-Label, Phase 2a, Multiple Basket Study. Lancet Oncol..

[112] Gupta R., Garrett-Mayer E., Halabi S., Mangat P.K., D’Andre S.D., Meiri E., Shrestha S., Warren S.L., Ranasinghe S., Schilsky R.L. (2020). Pertuzumab plus Trastuzumab (P+T) in Patients (Pts) with Colorectal Cancer (CRC) with ERBB2 Amplification or Overexpression: Results from the TAPUR Study. J. Clin. Oncol..

[113] Strickler J.H., Cercek A., Siena S., André T., Ng K., Van Cutsem E., Wu C., Paulson A.S., Hubbard J.M., Coveler A.L. (2023). Tucatinib plus Trastuzumab for Chemotherapy-Refractory, HER2-Positive, RAS Wild-Type Unresectable or Metastatic Colorectal Cancer (MOUNTAINEER): A Multicentre, Open-Label, Phase 2 Study. Lancet Oncol..

[114] Vernieri C., Milano M., Brambilla M., Mennitto A., Maggi C., Cona M.S., Prisciandaro M., Fabbroni C., Celio L., Mariani G. (2019). Resistance Mechanisms to Anti-HER2 Therapies in HER2-Positive Breast Cancer: Current Knowledge, New Research Directions and Therapeutic Perspectives. Crit. Rev. Oncol. Hematol..

[115] Siena S., Di Bartolomeo M., Raghav K., Masuishi T., Loupakis F., Kawakami H., Yamaguchi K., Nishina T., Fakih M., Elez E. (2021). Trastuzumab Deruxtecan (DS-8201) in Patients with HER2-Expressing Metastatic Colorectal Cancer (DESTINY-CRC01): A Multicentre, Open-Label, Phase 2 Trial. Lancet Oncol..

[116] RC48 Plus Tislelizumab, Low-Dose Capecitabine and Celecoxib for HER2-Positive Metastatic Colorectal Cancer—Full Text View—ClinicalTrials.gov. https://clinicaltrials.gov/ct2/show/NCT05578287?term=HER2&recrs=ab&cond=Colorectal+Cancer&draw=2&rank=1.

[117] Study of Tislelizumab Combined with DisitamabVedotin and Pyrotinib Maleate in HER2-Positive or Mutated Advanced Colorectal Cancer Who Failed Standard Therapy. https://clinicaltrials.gov/ct2/show/NCT05350917?term=HER2&recrs=ab&cond=Colorectal+Cancer&draw=2&rank=2.

[118] RC48-ADC Combined with Bevacizumab in HER2-Positive Advanced Colorectal Cancer. https://clinicaltrials.gov/ct2/show/NCT05785325?term=HER2&recrs=ab&cond=Colorectal+Cancer&draw=2&rank=3.

[119] A Safety and Efficacy Study of ZW25 (Zanidatamab) Plus Combination Chemotherapy in HER2-Expressing Gastrointestinal Cancers, Including Gastroesophageal Adenocarcinoma, Biliary Tract Cancer, and Colorectal Cancer. https://clinicaltrials.gov/ct2/show/NCT03929666?term=HER2&recrs=ab&cond=Colorectal+Cancer&draw=2&rank=4.

[120] To Evaluate IAH0968 in Combination with CAPEOX in HER2-Positive Metastatic Colorectal Cancer. https://clinicaltrials.gov/ct2/show/NCT05673512?term=HER2&recrs=ab&cond=Colorectal+Cancer&draw=2&rank=7.

[121] A Study of Tucatinib with Trastuzumab and mFOLFOX6 Versus Standard of Care Treatment in First-Line HER2+ Metastatic Colorectal Cancer—Full Text View—ClinicalTrials.gov. https://clinicaltrials.gov/ct2/show/NCT05253651?term=HER2&recrs=ab&cond=Colorectal+Cancer&draw=2&rank=8.

[122] Tucatinib Combined with Trastuzumab and TAS-102 for the Treatment of HER2 Positive Metastatic Colorectal Cancer in Molecularly Selected Patients, 3T Study. https://clinicaltrials.gov/ct2/show/NCT05356897?term=HER2&recrs=ab&cond=Colorectal+Cancer&draw=2&rank=9.

[123] Thein K.Z., Velcheti V., Mooers B.H.M., Wu J., Subbiah V. (2021). Precision Therapy for RET-Altered Cancers with RET Inhibitors. Trends Cancer Res..

[124] Nagasaka M., Brazel D., Baca Y., Xiu J., Al-Hallak M.N., Kim C., Nieva J., Swensen J.J., Spetzler D., Korn W.M. (2023). Pan-Tumor Survey of RET Fusions as Detected by next-Generation RNA Sequencing Identified RET Fusion Positive Colorectal Carcinoma as a Unique Molecular Subset. Transl. Oncol..

[125] Lin C., Wang S., Xie W., Chang J., Gan Y. (2015). The RET Fusion Gene and Its Correlation with Demographic and Clinicopathological Features of Non-Small Cell Lung Cancer: A Meta-Analysis. Cancer Biol. Ther..

[126] Pietrantonio F., Di Nicolantonio F., Schrock A.B., Lee J., Morano F., Fucà G., Nikolinakos P., Drilon A., Hechtman J.F., Christiansen J. (2018). RET Fusions in a Small Subset of Advanced Colorectal Cancers at Risk of Being Neglected. Ann. Oncol..

[127] FDA Approves Selpercatinib for Locally Advanced or Metastatic RET Fusion-Positive Solid Tumors. https://www.fda.gov/drugs/resources-information-approved-drugs/fda-approves-selpercatinib-locally-advanced-or-metastatic-ret-fusion-positive-solid-tumors.

[128] Subbiah V., Wolf J., Konda B., Kang H., Spira A., Weiss J., Takeda M., Ohe Y., Khan S., Ohashi K. (2022). Tumour-Agnostic Efficacy and Safety of Selpercatinib in Patients with RET Fusion-Positive Solid Tumours Other than Lung or Thyroid Tumours (LIBRETTO-001): A Phase 1/2, Open-Label, Basket Trial. Lancet Oncol..

[129] Lange A.M., Lo H.-W. (2018). Inhibiting TRK Proteins in Clinical Cancer Therapy. Cancers.

[130] Wang H., Li Z.-W., Ou Q., Wu X., Nagasaka M., Shao Y., Ou S.-H.I., Yang Y. (2022). NTRK Fusion Positive Colorectal Cancer Is a Unique Subset of CRC with High TMB and Microsatellite Instability. Cancer Med..

[131] Ratti M., Grizzi G., Passalacqua R., Lampis A., Cereatti F., Grassia R., Hahne J.C. (2021). NTRK Fusions in Colorectal Cancer: Clinical Meaning and Future Perspective. Expert Opin. Ther. Targets.

[132] Hua H., He W., Chen N., He Y., Wu G., Ye F., Zhou X., Li Y., Ding Y., Zhong W. (2022). Genomic and Transcriptomic Analysis of MSI-H Colorectal Cancer Patients with Targetable Alterations Identifies Clinical Implications for Immunotherapy. Front. Immunol..

[133] Drilon A., Laetsch T.W., Kummar S., DuBois S.G., Lassen U.N., Demetri G.D., Nathenson M., Doebele R.C., Farago A.F., Pappo A.S. (2018). Efficacy of Larotrectinib in TRK Fusion-Positive Cancers in Adults and Children. N. Engl. J. Med..

[134] Center for Drug Evaluation Research FDA Approves Larotrectinib for Solid Tumors with NTRK Gene Fusions. https://www.fda.gov/drugs/fda-approves-larotrectinib-solid-tumors-ntrk-gene-fusions-0.

[135] Doebele R.C., Drilon A., Paz-Ares L., Siena S., Shaw A.T., Farago A.F., Blakely C.M., Seto T., Cho B.C., Tosi D. (2020). Entrectinib in Patients with Advanced or Metastatic NTRK Fusion-Positive Solid Tumours: Integrated Analysis of Three Phase 1-2 Trials. Lancet Oncol..

[136] FDA Approves Entrectinib for NTRK Solid Tumors and ROS-1 NSCLC. https://www.fda.gov/drugs/resources-information-approved-drugs/fda-approves-entrectinib-ntrk-solid-tumors-and-ros-1-nsclc.

[137] Zheng G., Tseng L.-H., Haley L., Ibrahim J., Bynum J., Xian R., Gocke C.D., Eshleman J.R., Lin M.-T. (2019). Clinical Validation of Coexisting Driver Mutations in Colorectal Cancers. Hum. Pathol..

[138] Cecchini M., Sokol E., Vasan N., Pavlick D.C., Huang R.S.P., Pelletier M., Levy M.A., Pusztai L., Lacy J., Eder J.P. (2022). Molecular Characteristics of Advanced Colorectal Cancer and Multi-Hit PIK3CA Mutations. J. Clin. Oncol..

[139] André F., Ciruelos E., Rubovszky G., Campone M., Loibl S., Rugo H.S., Iwata H., Conte P., Mayer I.A., Kaufman B. (2019). Alpelisib for PIK3CA-Mutated, Hormone Receptor-Positive Advanced Breast Cancer. N. Engl. J. Med..

[140] Tabernero J., Van Geel R., Guren T.K., Yaeger R.D., Spreafico A., Faris J.E., Yoshino T., Yamada Y., Kim T.W., Bendell J.C. (2016). Phase 2 Results: Encorafenib (ENCO) and Cetuximab (CETUX) with or without Alpelisib (ALP) in Patients with Advanced BRAF-Mutant Colorectal Cancer (BRAFm CRC). J. Clin. Oncol..

[141] Juric D., Rodon J., Tabernero J., Janku F., Burris H.A., Schellens J.H.M., Middleton M.R., Berlin J., Schuler M., Gil-Martin M. (2018). Phosphatidylinositol 3-Kinase α-Selective Inhibition with Alpelisib (BYL719) in PIK3CA-Altered Solid Tumors: Results From the First-in-Human Study. J. Clin. Oncol..

[142] Ciombor K.K., Zemla T.J., Hubbard J.M., Jia J., Gbolahan O.B., Sousa A., Wilson L., Waechter B., Ou F.-S., Nixon A.B. (2023). A Phase II Single-Arm Study of the FGFR Inhibitor Pemigatinib in Patients with Metastatic Colorectal Cancer (mCRC) Harboring FGF/FGFR Alterations. J. Clin. Oncol..

[143] Hussung S., Akhoundova D., Sivakumar S., Kahraman A., Zoche M., Rechsteiner M., Angst F., Britschgi C., Töpfer A., Moch H. (2022). Frequency, Molecular Characteristics, and Therapeutic Targeting of ROS1 Oncogenic Fusions in Colorectal Cancer. J. Clin. Oncol..

[144] Hsiao S.-Y., He H.-L., Weng T.-S., Lin C.-Y., Chao C.-M., Huang W.-T., Tsao C.-J. (2021). Colorectal Cancer with EML4-ALK Fusion Gene Response to Alectinib: A Case Report and Review of the Literature. Case Rep. Oncol..

[145] Eng C., Jácome A.A., Agarwal R., Hayat M.H., Byndloss M.X., Holowatyj A.N., Bailey C., Lieu C.H. (2022). A Comprehensive Framework for Early-Onset Colorectal Cancer Research. Lancet Oncol..

[146] Akimoto N., Ugai T., Zhong R., Hamada T., Fujiyoshi K., Giannakis M., Wu K., Cao Y., Ng K., Ogino S. (2021). Rising Incidence of Early-Onset Colorectal Cancer—A Call to Action. Nat. Rev. Clin. Oncol..

[147] Pearlman R., Frankel W.L., Swanson B., Zhao W., Yilmaz A., Miller K., Bacher J., Bigley C., Nelsen L., Goodfellow P.J. (2017). Prevalence and Spectrum of Germline Cancer Susceptibility Gene Mutations Among Patients with Early-Onset Colorectal Cancer. JAMA Oncol..

[148] Sinicrope F.A. (2018). Lynch Syndrome-Associated Colorectal Cancer. N. Engl. J. Med..

[149] Mauri G., Sartore-Bianchi A., Russo A.-G., Marsoni S., Bardelli A., Siena S. (2019). Early-Onset Colorectal Cancer in Young Individuals. Mol. Oncol..

[150] Lundqvist E., Kuchinskaya E., Landerholm K., Assarsson J., Benckert A., Myrelid P., Haapaniemi S. (2022). Hereditary Evaluation and Genetic Counselling in Young Individuals with Colorectal Cancer in a Population-Based Cohort. Surg. Oncol..

[151] Khalessi Hosseini S.A., Morrissey C., Nakamura T., Becker D.J. (2020). Exploring the Cancer Genome Atlas (TCGA) for the Molecular Profile of Young Onset Colorectal Cancers. J. Clin. Oncol..

[152] Holowatyj A.N., Gigic B., Herpel E., Scalbert A., Schneider M., Ulrich C.M., MetaboCCC Consortium, ColoCare Study (2020). Distinct Molecular Phenotype of Sporadic Colorectal Cancers Among Young Patients Based on Multiomics Analysis. Gastroenterology.

